# Cholestasis in a Three Year-Old Child Following Abdominal Blunt Trauma: A Case Report

**DOI:** 10.5812/traumamon.12611

**Published:** 2013-10-13

**Authors:** Seyed Abdollah Mousavi, Hassan Karami

**Affiliations:** 1 Department of Pediatric Surgery, Mazandaran University of Medical Sciences, Sari, IR Iran; 2 Department of Pediatric Gastroenterology, Mazandaran University of Medical Sciences, Sari, IR Iran

**Keywords:** Trauma, Jaundice, Child, Bile Duct

## Abstract

**Introduction:**

Extra-hepatic bile duct injuries in children following blunt abdominal trauma are rare; early diagnosis and treatment are imperative for a good outcome. The purpose of this report is to describe the management of problems encountered in children with bile duct injuries following blunt abdominal trauma.

**Case Presentation:**

A three year-old girl presented with obstructive jaundice and vomiting following blunt abdominal trauma one month prior to referral. The child was sitting in her father’s lap when the accident occurred. She was then examined by an emergency physician to assess the cause of vomiting. An abdominal ultrasonography was performed and revealed dilatation of the common bile duct.

**Conclusions:**

To the best of our knowledge, this is the first report of bile duct injury following blunt trauma and its emergency management.

## 1. Introduction

Injuries to the gastrointestinal tract following blunt abdominal trauma continue to be a significant cause of morbidity and mortality in children. Optimal treatment of these injuries is associated withhigh prevalence of delayed diagnosis. On the other hand, traumatic obstructive jaundice following blunt trauma is extremely rare and a high clinical index of suspicion is necessary for early diagnosis. The purpose of this report is to describe the management of problems encountered in children with bile duct injuries following blunt abdominal trauma.

## 2. Case Presentation

**Figure 1.**
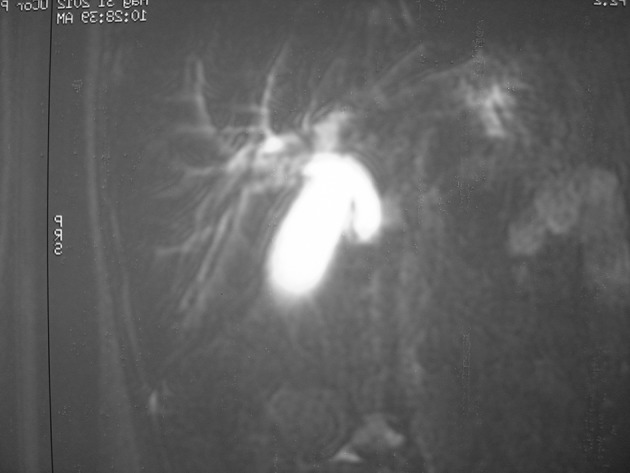
MRCP Showing Dilatation of the Hepatic Bile Ducts

A three year-old girl presented with abdominal pain and jaundice of one month duration. The pain was epigastric in origin, and worsened after eating. Her history was unremarkable except for blunt trauma following a car accident. The child was sitting on her father’s lap while he was driving whenthe accident occurred.After trauma the child was visited by a physician because of vomiting. When ultrasound and physical examination of the child was normal she was discharged and given metoclopramide drops. Over the next three days, she had nonbiliary vomiting which stopped thereafter. The child complained of occasional abdominal pain which was finally complicated by icterus. She appeared healthy and tolerated feeding well, although occasionally complaining of postprandial abdominal pain. She did not have a fever and her vital signs were normal. The physical examination showed a soft abdomen with no tenderness but revealed jaundice. The laboratory data results wereas follows: WBC = 12600 mm^3^, Hb = 12 g/dL, ESR = 25 mm/h, total bilirubin = 8.4 mg/dL, conjugated bilirubin = 6.9 mg/dL, aspartate aminotransferase (AST) = 559 IU/L, alanine aminotransferase (ALT) = 475 IU/L and alkaline phosphatase = 938 IU/L. A new ultrasound examination showed remarkable dilatation of the common bile duct to 8 mm with a terminal stricture. Barium meal showed a normal stomach and an open duodenum. Magnetic resonance cholangiopancreaticography (MRCP) demonstrated severe dilatation of the both intrahepatic and extrahepatic bile ducts (Figure 1). 

The patient was operated with a diagnosis of obstruction of the end of the common bile duct. The severe dilatation was obvious upon operation. The common bile duct was explored. A small catheter was passed with difficulty which pointed to severe stricture and fibrosis,not amenable to dilatation. The patient underwent choleducoduodenostomy. On the third day postoperatively, the patient started oral feeding. On the fifth day postoperatively, the serum bilirubin decreased significantly and the patient was discharged in good general condition. 

## 3. Conclusions

Visceral damage following blunt trauma occurs in less than 1% of the pediatric population (1). We reviewed the literature and found no report of the extrahepatic bile duct injury after blunt trauma in children. In two trauma centers in Nigeria, Chirdan etal. followed 19 children for 10 years and 21 children for 19 years and found only one duodenal hematoma after abdominal blunt trauma in each series (2). Galifer et al. (3) studied pediatric blunt trauma for seven years and found 16 cases of gastrointestinal damage with only one case with gall bladder complications. Lin (4) reported a ten year-old girl with biliary emesis after abdominal blunt trauma,diagnosed with duodenal hematoma, managed nonoperatively. Chein (5) introduced a six year-old boy with the same history and diagnosis that underwent laparoscopic surgery for vacating a hematoma. Clendenon et al. (6) studied 42 patients under 18 years of age with duodenal trauma and reported 33 cases with a history of blunt trauma. The diagnosis was mainly hematoma and 94% of patients underwent nonoperative management. Canty et al. (1) studied pediatric trauma for tenyears and reported that the most common organ damage after the blunt trauma was the small intestine followed by the duodenum. In a review article by Krishna et al. (7) with the aim of describing etiology for extrahepatic obstruction of bile ducts, 136 patients aged 1.5 to 15 years were evaluated. They found that in 93% of cases the cause of obstruction was either bile duct atresia or choleducal cyst. The following rare causes were responsible for obstruction in nine patients: 1) idiopathic benign nontraumatic inflammatory stricture; 2) idiopathic fibrosing chronic pancreatitis; 3) postcholecystectomy biliary stricture; 4) pseudocyst of pancreas; and 5) lymphoma with compression of the common bile duct. None of these patients had a history of abdominal blunt trauma. It is important to note the history in our patient. It was noted that icterus occurred one month after trauma for the first time. Considering the clinical course including abdominal pain and vomiting, it seemed that hematoma had formed after trauma in the duodenal wall. The bowel obstruction symptoms had resolved when icterus had emerged. We postulated that icterus in this setting may had result from: 1) extension of the hematoma to the papilla, organization, and fibrosis or 2) direct damage to the papilla following trauma. The dilated bile duct was the main reason for choleducoduodenostomy. We conclude that blunt abdominal trauma, should be considered among possible causes of acquired obstructive jaundice in previously healthy children.
